# Expressions of HuR, Methyl-HuR and Phospho-HuR in Endometrial Endometrioid Adenocarcinoma Are Associated with Clinical Features

**DOI:** 10.3390/ijms25020954

**Published:** 2024-01-12

**Authors:** Judicaël Hotton, Guillaume Gauchotte, Romane Mougel, Mégane Migliorini, Stéphanie Lacomme, Shyue-Fang Battaglia-Hsu, Mikaël Agopiantz

**Affiliations:** 1 Department of Gynecology and Obstetrics, CHRU de Nancy, Université de Lorraine, 54000 Nancy, France; judicael.hotton@reims.unicancer.fr; 2 INSERM U1256 NGERE, Université de Lorraine, 54500 Vandœuvre-lès-Nancy, France; g.gauchotte@chru-nancy.fr (G.G.); r.mougel@chru-nancy.fr (R.M.); megane.migliorini@univ-lorraine.fr (M.M.); shyue-fang.battaglia@univ-lorraine.fr (S.-F.B.-H.); 3 Department of Biopathology CHRU of Nancy, Institut de Cancérologie de Lorraine, BBB, CHRU de Nancy, Université de Lorraine, 54511 Vandœuvre-lès-Nancy, France; 4 Centre de Ressources Biologiques, BB-0033-00035, CHRU de Nancy, 54000 Nancy, France; s.lacomme@chru-nancy.fr; 5 Department of Reproductive Medicine, CHRU de Nancy, Université de Lorraine, 54000 Nancy, France

**Keywords:** endometrial adenocarcinoma, cancer, endometrium, immunohistochemistry, HuR, methyl-HuR, phospho-HuR

## Abstract

HuR regulates cytoplasmic mRNA stability and translatability, with its expression correlating with adverse outcomes in various cancers. This study aimed to assess the prognostic value and pro-oncogenic properties of HuR and its post-translational isoforms methyl-HuR and phospho-HuR in endometrial adenocarcinoma. Examining 89 endometrioid adenocarcinomas, we analyzed the relationship between HuR nuclear or cytoplasmic immunostaining, tumor-cell proliferation, and patient survival. HuR cytoplasmic expression was significantly increased in grade 3 vs. grade 1 adenocarcinomas (*p* < 0.001), correlating with worse overall survival (OS) (*p* = 0.02). Methyl-HuR cytoplasmic expression significantly decreased in grade 3 vs. grade 1 adenocarcinomas (*p* < 0.001) and correlated with better OS (*p* = 0.002). Phospho-HuR nuclear expression significantly decreased in grade 3 vs. grade 1 adenocarcinomas (*p* < 0.001) and non-significantly correlated with increased OS (*p* = 0.06). Cytoplasmic HuR expression strongly correlated with proliferation markers MCM6 (rho = 0.59 and *p* < 0.001) and Ki67 (rho = 0.49 and *p* < 0.001). Conversely, these latter inversely correlated with cytoplasmic methyl-HuR and nuclear phospho-HuR. Cytoplasmic HuR expression is a poor prognosis marker in endometrioid endometrial adenocarcinoma, while cytoplasmic methyl-HuR and nuclear phosphoHuR expressions are markers of better prognosis. This study highlights HuR as a promising potential therapeutic target, especially in treatment-resistant tumors, though further research is needed to understand the mechanisms regulating HuR subcellular localization and post-translational modifications.

## 1. Introduction

Endometrial cancer is the most frequent gynecologic cancer in high- and intermediate-income countries, and the fourth cancer in women after breast, colon, and lung cancers [1]. About 75% of endometrial cancers are diagnosed at an early stage, but more than two thirds of patients with advanced stages will die from cancer, despite improvements in diagnosis, surgery, and radiotherapy [2]. The discovery and characterization of new markers remain necessary in order to more accurately evaluate the prognosis and to develop specific treatments targeted to metastasis initiation and progression processes.

HuR (human antigen R; ELAVL1) is a 36 kDa ubiquitous protein, whose gene is located on chromosome 19 at position 19p13.2 [3]. HuR participates in the post-transcriptional regulation of mRNAs (messenger ribonucleic acids). In normal conditions, HuR is mainly located in the nuclei. It is thought to play a role in the alternative splicing of some mRNAs [4]. In survival and cell proliferation situations, in response to various stimuli, HuR binds in the nucleus to mRNA rich in AU (adenosine and uracile). The HuR-mRNA complex is then transported to the cytoplasm. Once HuR is linked to its target, it stabilizes the mRNA and protects it from rapid degradation by exonucleases. It then releases itself and returns rapidly to the nucleus after completing its stabilization process [5]. HuR enhances the expression of genes encoding proteins that increase cell proliferation, inhibit apoptosis, promote angiogenesis, reduce the immune response, and facilitate invasion [5]. In a study of 182 tumors of various origins [6], the level of expression of the HuR protein was largely increased in the cytoplasm of tumor tissues compared to non-tumor tissues, where it is preferentially poorly to moderately localized in the nuclei [7]. These data have been confirmed in other studies, and the cytoplasmic overexpression was found to be correlated with higher grade, larger tumors, and high mitotic activity in breast and ovarian cancers, highlighting the potential diagnostic, prognostic, and therapeutic value of the protein in gynecologic cancers [8,9]. Our team confirmed these features in meningioma and highlighted the prognostic value of methyl-HuR in lung cancer [10,11].

To date, only one study has evaluated HuR expression in 109 cases of endometrioid endometrial adenocarcinomas [12]. The authors reported that cytoplasmic overexpression of HuR was significantly associated with higher grades and stages. No study was carried out regarding the post-translational analysis of HuR in this cancer.

The main objective of this retrospective study was to evaluate the prognostic value of HuR and two of its post-translational variants, methyl-HuR (mHuR) and phospho-HuR (pHuR), in a cohort of 89 cases of endometrioid endometrial adenocarcinomas. We have also studied the correlation between staining for HuR, mHuR, or pHuR expressions and Ki67 and MCM6 proliferation markers.

## 2. Results

### 2.1. Population Characteristics

Clinical and histological data are summarized in our previous publication [13]. Mean age at diagnosis was 66.8 ± 11.0 years. Thirty-one percent of patients were obese (BMI > 30 kg/m^2^). The average age at menopause was 51.9 ± 3.6 years. The diagnosis was made in 82% of cases following the occurrence of post-menopausal bleeding. The average follow-up time was approximately 47 months. The progression rate was about 9%, with 11.2% being deaths.

All included cases were endometrial endometrioid adenocarcinomas. Majority of cases were classified as stage 1 according to the FIGO classification (79.8%). There was an invasion of the myometrium ≥50% in 40.4% of cases. Lymphovascular space invasions (LVSI) were found in 20.2% of cases. In total, 4 of the 54 pelvic lymph node dissections were positive (7.4%). Distant metastases were found throughout the follow-up in 8 cases (9%).

There was no statistically significant association between histological grade and survival, either overall (OS: log-rank test, *p* = 0.1) or in terms of progression-free survivals (PFS: log-rank test, *p* = 0.5). Univariate Cox model was significant for OS (*p* = 0.039) but not for PFS (*p* = 0.26) (Table 1).

### 2.2. HuR Expression in Endometrioid Endometrial Cancer

**Figure 1:**
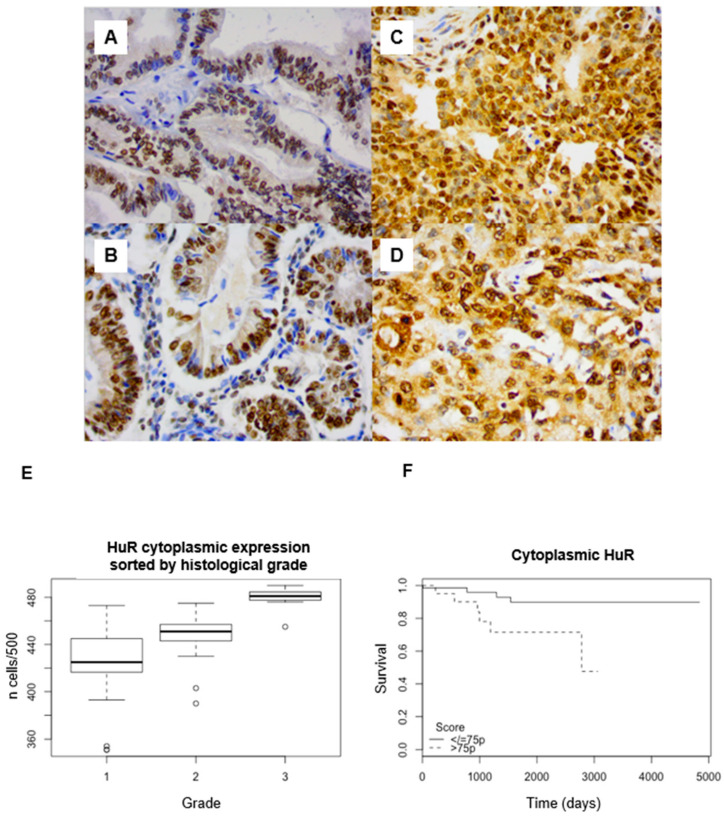
(**A**–**D**) HuR immunohistochemical staining in endometrial endometrioid adenocarcinoma. Weak cytoplasmic staining in a case of grade 1 adenocarcinoma ((**A**) ×200 magnification; (**B**) ×400). Strong cytoplasmic staining in a case of grade 3 adenocarcinoma ((**C**) ×200 magnification; (**D**) ×400). (**E**) Expression of cytoplasmic HuR sorted by histological grade (*p* < 0.001). Strong nuclear staining in both cases. (**F**) Survival analyses. Kaplan–Meier curves with log-rank tests. Correlation between HuR cytoplasmic expression and overall survival (*p* = 0.021) (threshold: 75pc).

HuR expression was nuclear and cytoplasmic, predominantly cytoplasmic, with an average of 88.4% (95% confidence interval (95CI): 87.2–89.5) positive cells. The labeling index (LI) was rather heterogeneous, both for nuclear and cytoplasmic staining (Figure 1 A–D).

There was a significant cytoplasmic overexpression of HuR in grade 3 (mean: 95.9%, 95CI: 94.6–97.2) compared to grade 1 (mean: 85%, 95CI: 83.2–86.8) (*p* < 0.001) and grade 2 (mean: 89.6%, 95CI: 88.6–90.6) adenocarcinomas (Figure 1E). Dunn’s post hoc test showed a significant difference between each pair of grades (*p* < 0.001).

HuR cytoplasmic LI was negatively correlated with OS, both with the log-rank test (threshold: 75th percentile, *p* = 0.02) (Figure 1F) and the univariate Cox model, with a hazard ratio (HR) = 4 (95CI: 1.1–14.2, *p* = 0.03; 75th percentile), without correlation with PFS (Table 1). In multivariate Cox regression, in a model combining grade and stage, HuR cytoplasmic expression remained significantly correlated with shorter OS (*p* = 0.03).

No significant difference between nuclear staining for HuR and grade (*p* = 0.33) or survival was found (OS, *p* = 0.763, PFS; *p* = 0.187).

### 2.3. Post-Translational Modified HuR Expression

**Figure 2:**
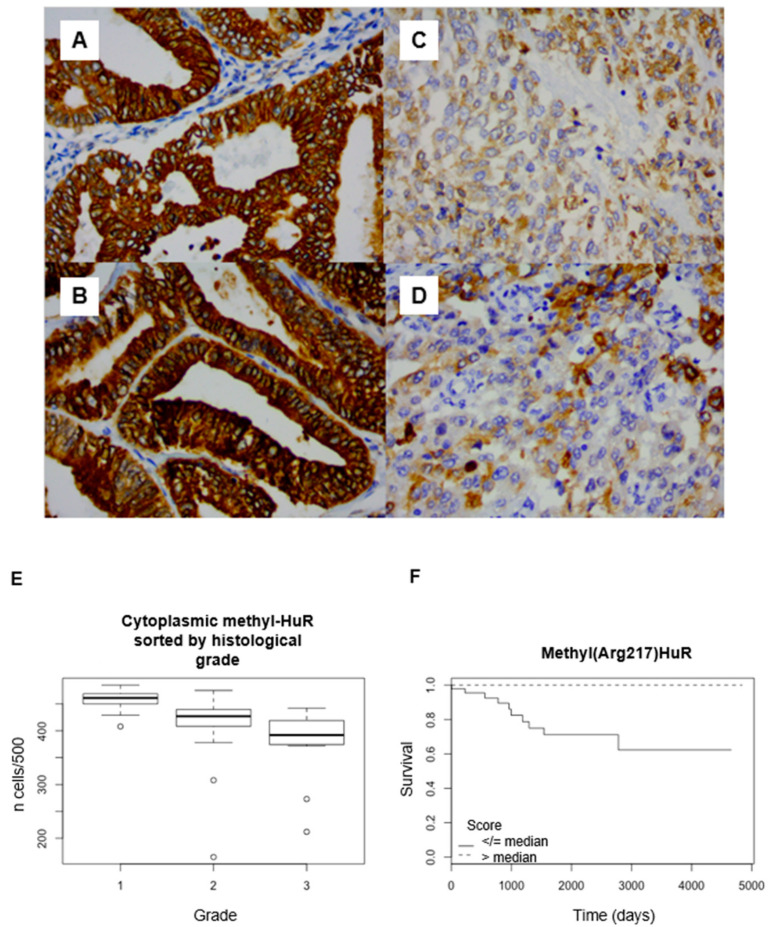
(**A**–**D**) Methyl-HuR immunohistochemical staining in endometrial endometrioid adenocarcinoma. Strong cytoplasmic staining in a case of grade 1 adenocarcinoma ((**A**) ×200 magnification; (**B**) ×400). Moderate cytoplasmic staining in a case of grade 3 adenocarcinoma ((**C**) ×200 magnification; (**D**) ×400). (**E**) Expression of cytoplasmic methyl-HuR sorted by histological grade (*p* <0.001). (**F**): Survival analyses. Kaplan–Meier curves with log-rank tests. Correlation between methyl-HuR cytoplasmic expression and overall survival (*p* = 0.003) (threshold: median).

mHuR was mainly expressed in the cytoplasm (mean: 85.8%, 95CI: 83.6–87.9). This expression sometimes appeared heterogeneous (Figure 2A–D). mHuR was significantly more expressed in the cytoplasm of lower-grade tumors (*p* < 0.001) and in lower stages (*p* = 0.004) (Figure 2E). Additionally, the expression of mHuR was significantly lower in cases of LVSI (81.3% vs. 86.9%, *p* = 0.01), lymph node invasion (78.5% vs. 86%, *p* = 0.01), or metastasis (74.7% vs. 86.9%, *p* = 0.03). Cytoplasmic overexpression of mHuR was positively correlated with OS (log-rank, threshold: median; *p* = 0.003) and PFS (log-rank, threshold: 25th percentile; *p* = 0.03) (Figure 2F). With univariate Cox analyses, cytoplasmic mHuR LI was significantly correlated with OS (*p* = 0.001) and PFS (*p* = 0.007). With the multivariate Cox model, cytoplasmic mHuR expression was correlated with survival (*p* = 0.025), independently from grade and stage. No data was significant when considering nuclear mHuR expression (Table 1).

**Figure 3:**
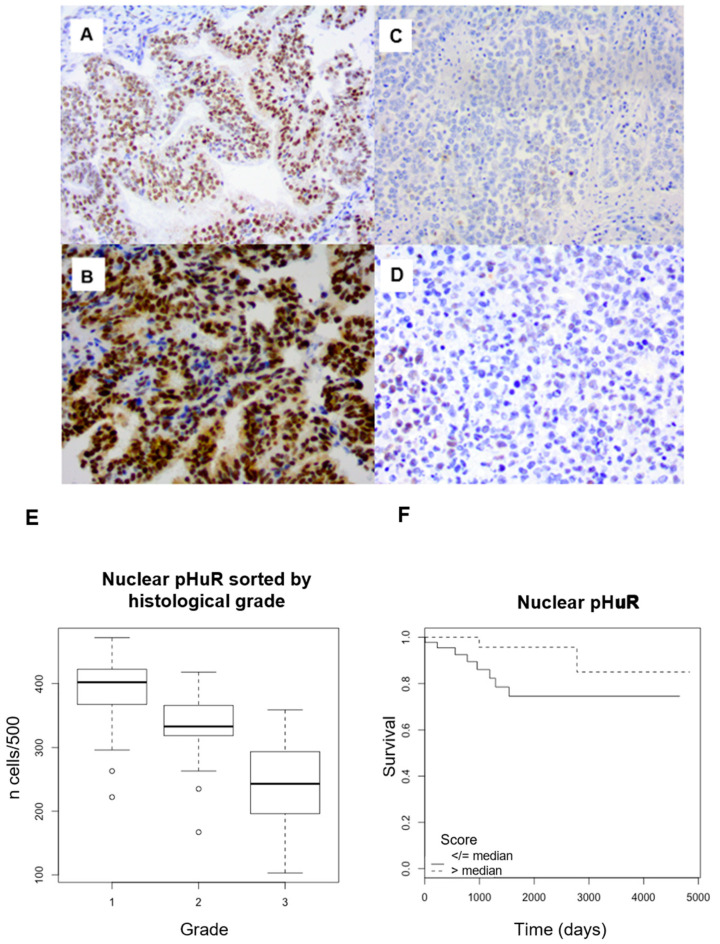
(**A**–**D**) Phospho-HuR immunohistochemical staining in endometrial endometrioid adenocarcinoma. Strong nuclear staining in a case of grade 1 adenocarcinoma ((**A**) ×200 magnification; (**B**) ×400). Weak nuclear staining in a case of grade 3 adenocarcinoma ((**C**) ×200 magnification; (**D**) ×400). (**E**) Nuclear expression of phospho-HuR sorted by histological grade (*p* < 0.001). (**F**) Survival analyses. Kaplan–Meier curves with log-rank tests. Correlation between phospho-HuR nuclear expression and overall survival (*p* = 0.06) (threshold: median).

pHuR expression was predominantly nuclear with an average of 69.4% (95CI: 66.4–72.5) (Figure 3A–D). pHuR nuclear expression was inversely correlated with histological grade, with mean values of 78.4% (95CI: 74.9–81.8) in grade 1, 66.3% (95CI: 63.2–69.3) in grade 2, and 48.9% (95CI: 38.1–59.8) in grade 3 (*p* < 0.001) (Figure 3E). Its expression had no statistical association with the tumor stage (Kruskal–Wallis test: *p* = 0.2). There was a significant decrease in the expression of pHuR in the nuclei in the case lymph node invasion (53.5% vs. 68.9%, *p* = 0.009), as well as in the presence of LVSI (60.4% vs. 71.7%, *p* = 0.009). In terms of OS, there was a non-significant trend to a positive correlation with nuclear expression of pHuR (log-rank, threshold: median; *p* = 0.06) (Figure 3F). There was no significant correlation with PFS (log-rank test: *p* = 0.208) (Table 1). 

HuR cytoplasmic expression was inversely correlated with mHuR cytoplasmic expression (rho = −0.41 and *p* < 0.001) and pHuR nuclear expression (rho = −0.44 and *p* < 0.001), these latter two varying significantly in the same direction (rho = 0.52 and *p* < 0.001).

### 2.4. Proliferation Markers Correlation with HuR Expression

The expression levels of MCM6 and Ki67 were previously published by our team [13]. Cytoplasmic expression of HuR was strongly correlated with the expression levels of MCM6 (rho = 0.59 and *p* < 0.001) and Ki67 (rho = 0.49 and *p* < 0.001). In contrast, they were inversely correlated with cytoplasmic mHuR (rho = −0.31 and *p* < 0.001 for MCM6; rho = −0.34 and *p* < 0.001 for Ki-67) and with nuclear pHuR (rho = −0.19 and *p* < 0.001 for MCM6; rho = −0.27 and *p* < 0.001 for Ki-67).

### 2.5. Inter-Observer Reproducibility

The intra-class correlation coefficient (ICC) was good for both cytoplasmic HuR (ICC = 0.56, 95CI: 0.34–0.73) and nuclear HuR LI (ICC = 0.58, 95CI: 0.37–0.74). Intra-class correlation coefficient was excellent for cytoplasmic mHuR (ICC = 0.88; 95CI: 0.81–0.93) and nuclear mHuR expression (ICC = 0.95; 95CI: 0.92–0.97), with similar results for pHuR (nuclear: ICC = 0.92; 95CI: 0.86–0.95; cytoplasmic: ICC = 0.98, 95CI: 0.97–0.99).

The intra-class correlation coefficients for the MCM6 and Ki-67 proliferation markers were also very high (ICC = 0.8 for both).

## 3. Discussion

### 3.1. HuR Is Overexpressed in Endometrial Adenocarcinoma

HuR upregulation is known to play a central role in cancer initiation and progression. It is an RNA-binding protein (RBP) which, by its three RNA recognition motifs (RRM) domains, specifically recognizes adenosine and uracile (AU)-rich sequences located in untranslated 3’ region of pro-oncogenic RNA messenger, stabilizing them and modulating their translation [14]. The RRM2 and RRM3 domains are separated by a “hinge region” containing HuR nuclear shuttling (HNS) domain, which is involved in its nucleo-cytoplasmic transport [15]. Being mainly located in the nucleus, the transport of HuR to the cytoplasm, via the HNS domain, appears essential to ensure its regulatory functions [16]. Thus, HuR has been shown to be highly expressed in malignant tumors and capable of binding to the 3′-UTR of cytokine and/or angiogenic factor mRNAs [11,17]. Furthermore, the HuR level is positively correlated with tumor grade in breast carcinoma [9]. Its cytoplasmic expression is an established prognostic factor for invasive ductal breast carcinoma, which is an estrogen-dependent cancer [18]. Although HuR aberrant or over-expression has been reported in a wide range of other cancer types, including ovarian [19], gastric [20], colon [21], and lung [11] adenocarcinoma, very limited information is available regarding HuR expression in endometrial adenocarcinoma [12]. In the present study, we provide evidence that the level of cytoplasmic HuR increases with tumor grade and is correlated with worse overall survival. These results support the only previous study that showed that cytoplasmic HuR expression was more frequent in poorly differentiated carcinomas, in an advanced stage, and associated with positive estrogen receptor alpha (ER-α) expression [12]. In this study, after HuR short hairpin RNAs transfection into Ishikawa cells, the ER-α protein level was decreased, and the decreased expression of HuR resulted in the inhibition of proliferation and induced apoptosis [12]. These results showed that HuR could be a causal factor of ER-α regulation and may induce the hormone-dependent endometrial carcinoma. They are concordant with a study showing that HuR immunoreactivity was significantly lower in the endometrium of early proliferative and late secretory phases, compared to the mid-late proliferative and early-mid secretory phases [22].

### 3.2. Cell Proliferation Markers Are Correlated with HuR Overexpression

As HuR targets multiple proteins involved in cell cycle regulation, such as cyclins, p21, and p27, HuR overexpression is expected to be correlated with higher proliferation index [5]. In our study, cytoplasmic expression of HuR was strongly correlated with the expression levels of two markers of proliferation, MCM6 and Ki67. These data are concordant with our previous studies showing the prognostic value of the proliferation markers MCM6 and Ki-67 in endometrial adenocarcinoma [13], lung cancer [11], and meningioma [23]. 

### 3.3. Significance of mHuR Expression

Cytoplasmic localization of HuR seems necessary to exert its functions, notably for the stabilization of target mRNA. Mechanisms involved in HuR subcellular localization may be linked to different types of post-transcriptional modifications, including phosphorylation and methylation in its HNS (HuR nucleo-cytoplasmic shuttling sequence) domain [24].

In our study, mHuR cytoplasmic expression was significantly decreased in grade 3 compared to grade 1 adenocarcinomas, was correlated with better OS, and was inversely correlated with the expression levels of markers of proliferation MCM6 and Ki67. It should be noted that although significant, the impact of mHuR on overall survival (HR = 0.99) is much less than that of HuR (HR = 4). HuR methylation may promote its passage into the cytoplasm and/or inhibit its reintroduction into the nucleus and be involved in carcinogenesis. This phenomenon is dependent on CARM-1 (coactivator-associated arginine methyltransferase 1), an arginine methyl transferase, which acts mainly on arginine at position 217 [25]. Its position on the HNS domain suggests its involvement in HuR’s passage through the nuclear membrane. It appeared that mHuR level was significantly decreased in tumor tissues [26]. Another study in a cohort of 190 non-small-cell bronchial adenocarcinomas showed a significant correlation between cytoplasmic expressions of HuR and mHuR, as well as a significant correlation between mHuR expression and lower-grade lung cancers. This expression was inversely correlated with the proliferation index. These data suggest that the non-methylated form of HuR would have an opposite effect to its methylated form methyl(R217)HuR, explaining why the overexpression of mHuR would be associated with a rather good prognosis, probably by decreasing the interactions between HuR and its mRNA targets, and thus repressing tumor cell proliferation [11]. 

### 3.4. Significance of pHuR Expression

Other regulatory mechanisms may also influence the cytoplasmic localization of HuR [24]. Phosphorylation of HuR on a serine in position 221 of the HNS domain would cause a decrease in the nucleo-cytoplasmic passage of HuR and thus a decrease in its action. This phenomenon would be under the dependence of the protein kinase C (PKC) [27]. In our study, pHuR nuclear expression was significantly decreased in grade 3 compared to grade 1 adenocarcinomas, was correlated with better OS, and was inversely correlated with the expression levels of markers of proliferation MCM6 and Ki67. PKCα interacts with HuR in the nucleus to phosphorylate S158 and S221, leading to ATP-dependent HuR cytoplasmic translocation, but it is not critical for binding to RNA in the case of S221 phosphorylation [28]. The impact of HuR phosphorylation by PKC in carcinogenesis is complex and site dependent. Interestingly, in breast cancer cells undergoing apoptosis in response to doxorubicin, HuR was reported to be phosphorylated by PKCδ at S221 and S318 [29].

### 3.5. Clinical Perspectives

Endometrioid-type endometrial adenocarcinoma, the most frequent histological type of uterine corpus cancer, has a relatively good prognosis and is in most of cases diagnosed at an early stage [30]. New approaches and treatments are needed, especially for high histological grade, advanced tumor stage, TP53 mutated form (which is associated with HuR overexpression in breast cancer [31]), and in the case of microsatellite instability/mismatch repair (MMR) deficiency, which are associated with a poor prognosis [32,33,34]. HuR is a molecular target for cancer therapeutics and immune-related disorders [35]. Small molecules, RNA interference and nanoparticle-based drug delivery approaches targeting HuR have showed anticancer activity [35]. Some small molecules can block HuR association with target mRNAs or target HuR translocation (including in MCF7 breast cancer cells) or HuR trafficking [36,37,38]. For example, pharmacological inhibition of PKCδ by rottlerin abolished the S318 phosphorylation of HuR. Its binding to target mRNAs in colon cancer cells demonstrated that targeting PKC isoforms may alter HuR functions in cancer [39]. Targeting HuR or the molecules involved in its post-translational regulation seems to be very promising therapeutic approach and deserves further studies.

## 4. Materials and Methods

### 4.1. Study Population

Eighty-nine consecutive cases of endometrial endometrioid adenocarcinoma were retrospectively retrieved from the tissue biobank of the Department of Pathology (CHRU) in Nancy (France), between 2000 and 2008.

Histological grade according to WHO classification, myometrial invasion, lymphovascular space invasion, and lymph node invasion were controlled by an experienced pathologist [40]. A representative block of formalin-fixed paraffin-embedded tissues for each case was selected, corresponding to the areas of highest tumor density. Five µm cuts were made and stained by hematoxylin, eosin, and safranin (HES).

Clinical data were collected from the files of the Gynecological Surgery Department of CHRU in Nancy and the Radiotherapy Department of the Lorraine Cancer Institute (Vandoeuvre-lès-Nancy), including, if available, data describing patient age, progression-free survival (PFS) time, and overall survival (OS) time.

### 4.2. Immunohistochemistry

Five µm paraffin sections were immersed in a 10 mM sodium citrate buffer (pH 6) for 20 min at 97 °C for dewaxing and antigen retrieval. Staining for HuR, mHuR, and pHuR was achieved using primary anti-HuR (1/3000; mouse monoclonal, Santa Cruz Biotechnology, Heidelberg, Germany), anti-mHuR (1/2000; methyl(Arg217)HuR rabbit polyclonal, homemade), and anti-pHuR (1/200; phospho(Ser221)HuR rabbit polyclonal, Abcam, Cambridge, UK) antibodies. Specificity of methyl(R217)HuR homemade antibody was confirmed by Western blotting (in meningioma cases), showing the expected 36 kDa band, and with a specific inhibition test with the peptide H-HHQAQR(AsymMe2)FRFSP-NH2 (Eurogentec, Seraing, Belgium). Staining for MCM6 and Ki-67 was achieved using primary anti-MCM6 (1/400; goat polyclonal, Santa Cruz Biotechnology, Heidelberg, Germany) and anti-Ki-67 (1/200; mouse monoclonal, MIB-1, Dako Cytomation, Glostrup, Denmark) antibodies. Immunohistochemistry was performed with Dako Autostainer Plus (Dako Cytomation, Glostrup, Denmark) and Flex+ Envision revelation system (Dako Cytomation, Glostrup, Denmark). Negative controls (without primary antibody) were used throughout the experiment.

Ki67 and MCM6 expressions were analyzed quantitatively, corresponding to the percentage of marked cells, by counting 500 cells in hotspots. For HuR, mHuR, and pHuR, nuclear and cytoplasmic scores were obtained, corresponding to the percentage of marked cells, by counting 100 cells five times in five contiguous fields (500 cells in total), in hotspots.

### 4.3. Statistical Analysis

The statistical analysis was performed using R freeware (R Foundation for Statistical Computing, Vienna, Austria; version 4.2.3).

The quantitative variables were described by their mean value with standard deviation or by their frequency of distribution. As variables did not follow a normal distribution (Kolmogorov–Smirnov test), all clinical and immunohistochemical data were compared by the non-parametric Wilcoxon test for variables with two modalities, and by the Kruskal–Wallis test for those with more than two modalities. The results were then compared in each group using Dunn’s post hoc test. Spearman correlation coefficient was used to assess the correlation between the different immunohistochemical markers. The concordance between the two raters was measured for each immunohistochemical marker using the intra-class correlation coefficient for the quantitative variables.

Overall survival (OS) and progression-free survival (PFS) were estimated by the Kaplan–Meier method and represented in days. Survival analyses were performed using the log-rank test and Cox model with univariate regression. Only the variables that were significantly correlated with univariate Cox regression modalities were analyzed with the multivariate Cox model; a goodness-of-fit test was performed. 

For all tests performed, a *p*-value of 0.05 or less was considered statistically significant.

## 5. Conclusions

To the best of our knowledge, this study was the first to assess the prognostic value of cytoplasmic HuR, cytoplasmic mHuR, and nuclear pHuR expressions in endometrioid endometrial adenocarcinoma, showing a significant correlation with grade and survival, with a very good reproducibility. HuR is a promising potential therapeutic target for use in tumors refractory to standard therapies. Identification and mechanistic characterization of the HuR post-translational modifications are essential. However, the main weakness of our study is the small size of our cohort and the low number of events (progression and death), which impact the interpretation of survival results. Larger-scale clinical complementary studies and mechanistic studies are needed to enhance these preliminary results.

## Figures and Tables

**Table 1:** Log rank and univariate Cox survival analyses.

	PFS				OS			
Variables	LOG RANK		Cox Univariate Analyses		Log Rank		Cox Univariate Analyses	
	*p*-Value	HR	95% CI	*p*-Value	*p*-Value	HR	95% CI	*p*-Value
**WHO grade**	0.5	1.767	0.658–4.742	0.26	0.1	2.548	1.047–6.204	0.039 *
**HuR expression**								
Nuclear	0.187	0.356	0.072–1.168	0.207	0.763	1.21	0.35–4.187	0.763
**Cytoplasmic**	0.7	0.775	0193–3.109	0.72	0.021 *	**4**	**1.1–14.2**	0.033 *
**m-HuR expression**								
Nuclear	0.8	1.147	0.287–4.589	0.846	0.9	1.095	0.317-3.783	0.886
**Cytoplasmic**	0.03 *	**0.991**	**0.984–0.998**	0.007 *	0.003 *	**0.989**	**0.983–0.996**	0.001 *
**p-HuR expression**								
**Nuclear**	0.208	0.372	0.075–1.845	0.226	**0.06**	**0.257**	**0.054–1.219**	**0.087**
Cytoplasmic	0.741	1.269	0.317–5.082	0.736	0.7	1.232	0.356–4.265	0.742

CI: confidence interval; HR: hazard ratio; OS: overall survival; PFS: progression-free survival; WHO: World Health Organization; * *p* < 0.05.

## Data Availability

Data are contained within the article.
